# A Dose–response relationship between severity of disc degeneration and intervertebral disc height in the lumbosacral spine

**DOI:** 10.1186/s13075-015-0820-1

**Published:** 2015-10-23

**Authors:** Andrew J. Teichtahl, Donna M. Urquhart, Yuanyuan Wang, Anita E. Wluka, Stephane Heritier, Flavia M. Cicuttini

**Affiliations:** Department of Epidemiology and Preventive Medicine, School of Public Health and Preventive Medicine, Monash University, Alfred Hospital, 99 Commercial Rd, Prahran, Melbourne, VIC 3004 Australia; Baker IDI Heart and Diabetes Institute, Commercial Road, Melbourne, VIC 3004 Australia

**Keywords:** Lumbar, Intervertebral disc, Disc degeneration, Height

## Abstract

**Introduction:**

Varied definitions of disc pathology exist in the literature. Magnetic Resonance Imaging (MRI) classification systems incorporate several qualitative features including disc appearance, the distinction between the nucleus and the annulus, signal intensity and intervertebral disc height. The lack of a continuous measure has made it difficult to sensitively examine degenerative disc disease. This study sought to examine the relationship between disc degeneration and intervertebral disc height.

**Methods:**

72 community-based individuals not selected for low back pain had MRI from which the presence of lumbosacral disc degeneration was identified using the Pfirrmann grading system, and intervertebral disc height was measured.

**Results:**

At each lumbosacral level, with higher grade of disc degeneration, intervertebral disc height was reduced (all p ≤ 0.003). Results remained unchanged when grade 5 disc degeneration, which necessitated a collapsed disc space, was excluded from analyses (all p ≤ 0.03). To quantify these associations, at each lumbosacral level, for every grade increase in disc degeneration, there was a reduction in intervertebral disc height, after adjusting for age, gender, Body mass index and smoking history (β range from −0.98 mm to −1.60 mm, 95 % CI range from −2.37 to −0.31, all p ≤ 0.005).

**Conclusion:**

This study has demonstrated a negative dose–response relationship between increasing severity of disc degeneration with a reduction in intervertebral disc height. Although the assessment of disc degeneration incorporates a number of qualitative measures, these data substantiate the utility of intervertebral disc height as a quantitative and continuous outcome measure in epidemiological studies, and potentially clinical practice.

## Significance and innovation

No continuous measure of lumbar disc degeneration exists. Identifying a reliable and easily accessible measure would enable a sensitive means for assessing disease pathogenesis. This study provides the first evidence that intervertebral disc height, a continuous, reliable and easily accessible measure, is a sensitive determinant of lumbar disc degeneration.

## Introduction

Lumbosacral disc degeneration is common, with approximately one third of asymptomatic young individuals exhibiting this feature on magnetic resonance imaging (MRI) [1]. Moreover, the presence of disc degeneration in the lumbar spine signifies a two-fold increased risk of chronic low back pain [2, 3], making it an important clinical endpoint in epidemiological studies.

**Fig. 1:**
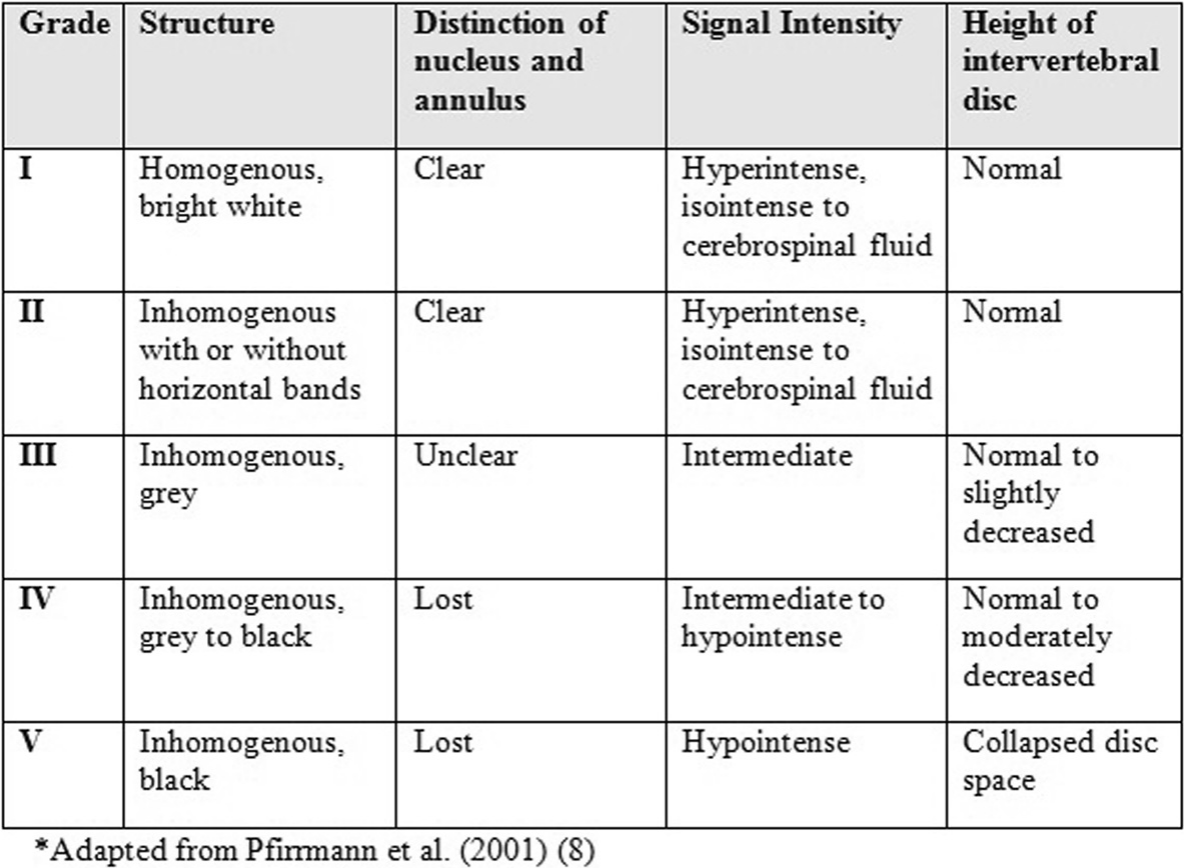
Pfirrmann grading of lumbosacral disc degeneration. Adapted from Pfirrmann et al. (2001) [8]

There are varied definitions in the literature of what constitutes a degenerative disc. In histological studies these include chondrocyte proliferation, mucous degeneration, cell death, tear and cleft formation and granular changes [4], while macroscopic grading systems incorporate changes in the nucleus, annulus, endplate and vertebral body [5]. Radiographic studies classify disc degeneration by varying grades of joint space narrowing, osteophytes and endplate sclerosis [6, 7]. In a systematic review of existing grading systems for lumbar disc degeneration, Pfirrmann’s method (2001) [8] was endorsed as a valid and reliable method of assessing intervertebral disc degeneration using MRI [9]. The Pfirrmann system uses a number of qualitative MRI features including the appearance of the disc structure, the distinction between the nucleus and the annulus, the signal intensity and the intervertebral disc height, to give a 5-point grading system [8] (Fig. 1). Nevertheless, there is no continuous measure of disc degeneration in the literature, making it difficult to sensitively examine change in degenerative disc disease. As intervertebral disc height can be readily assessed quantitatively from MRI, this study sought to examine whether there was a dose–response relationship between the severity of disc degeneration assessed using the Pfirrmann method, and intervertebral disc height.

## Methods

### Participants

Seventy-two community-based individuals were recruited through local media and weight loss clinics without reference to whether they had or did not have low back pain. Participants were excluded if they had a history of malignancy, significant systemic condition (e.g., cerebrovascular accident, movement disorder, or connective tissue disease), contraindication to MRI or inability to understand English. Participants gave written informed consent. The study was approved by the Human Research Ethics Committees of the Alfred Hospital and Monash University.

### Magnetic resonance imaging

MRI was performed using a 3.0-T magnetic resonance unit (MAGNETOM Verio, A Tim System; Siemens, Erlangen, Germany). The participant was positioned in the supine position and the following scans were performed: 1) sagittal T1 images from vertebra T12 to the sacrum (repetition time 670 ms, echo time to echo 12 ms, slice thickness 4 mm); 2) sagittal T2 images from vertebra T12 to the sacrum (repetition time 3000–3600 ms, echo time 87–114 ms, slice thickness 4 mm).

#### Degenerative intervertebral disc assessment

Intervertebral disc degeneration was assessed from T2-weighted sagittal images based on the Pfirrmann method [8] (Fig. 1). The measurement was performed by one assessor who was trained by a radiologist experienced in musculoskeletal MRI. Images were reassessed 1 week apart, blinded to the characteristics of the participants. The intra-rater reliability of the disc degeneration measures at each vertebral level was high, with intra-class correlation coefficients (ICCs) ranging from 0.88 to 0.94.

#### Intervertebral disc height

Intervertebral disc height in the lumbosacral spine was measured on T1-weighted mid-sagittal MR images from the middle of the superior border of the disc to the middle of the inferior border of the disc with the inclusion of both endplates. One trained observer was taught to measure disc height by a radiologist experienced in musculoskeletal MRI, and measured the disc height in duplicate, 1 week apart, blinded to the characteristics of the participants. The intra-rater reliability of the disc height measures at each vertebral level was high, with ICCs ranging from 0.94 to 0.98.

### Anthropometric data

Height was measured to the nearest 0.1 cm using a stadiometer. Weight was measured to the nearest 0.1 kg using a single pair of electronic scales. Body mass index (BMI) (kg m^−2^) was calculated.

### Smoking history

Participants were asked whether they were past/present smokers or had never smoked.

### Chronic pain and disability

The chronic pain grade questionnaire was administered at the time of MRI in 2011–2012 to obtain information on low back pain intensity over the past 6 months. The chronic pain grade questionnaire is a reliable and valid instrument for use in population surveys of low back pain [10, 11]. The questionnaire includes seven questions from which a pain intensity and disability subscale score are calculated. Subscale scores for pain intensity and disability are combined to calculate a chronic pain grade that enables classification of chronic pain into five hierarchical categories: grades 0 (no pain) to 4 (high disability, severely limiting) as previously described [10, 11]. High intensity pain/disability was defined as being of either grade 2 (low disability but high intensity), grade 3 (high disability, moderately limiting) or grade 4 (high disability, severely limiting).

### Statistical analyses

For each grade of disc degeneration, the *F* test was used to determine whether the null hypothesis that the mean intervertebral disc heights were homogenous, could be rejected. Subgroup analyses were performed excluding grade-5 disc degeneration, as this represented the most severe degeneration with a collapsed intervertebral disc. Linear regression analyses were used to determine the dose–response relationship between the severity of disc degeneration and intervertebral disc height for each lumbosacral disc, adjusted for age, gender, BMI and smoking history. Post hoc analyses were performed to compare the average disc height for people with and without high pain and or disability. A *p* value less than 0.05 (two-tailed) was regarded as statistically significant. All analyses were performed using the SPSS statistical package (standard version 20.0 SPSS, Chicago, IL, USA).

## Results

The characteristics of the 72 participants are shown in Table 1. The mean (± standard deviation) age of the cohort was 48.7 (±8.3) years, comprising 49 (68.1 %) female participants, and the participants were on average, overweight (BMI 29.2 kg m^−2^ ± 7.9 kg m^−2^). Forty-three percent of participants were past or present smokers. The frequency of each grade of disc degeneration for each corresponding lumbosacral level is shown in Table 1, with an overall representation of more severe grades of disc degeneration apparent at vertebrae L4/5 and L5/S1.

**Table 1:** Subject characteristics (*n* = 72)

Characteristic	Value				
Age, years	48.7 (8.3)				
Gender, n (%) female	49 (68.1)				
BMI, kgm^−2^	29.2 (7.9)				
Smoking, past/present, n (%)	31 (43.1)				
Disc degeneration grade, n (%)	1	2	3	4	5
L1/2	0 (0)	51 (70.8)	18 (25.0)	2 (2.8)	1 (1.4)
L2/3	0 (0)	37 (51.4)	27 (37.5)	8 (11.1)	0 (0)
L3/4	0 (0)	25 (34.7)	36 (50.0)	10 (13.9)	1 (1.4)
L4/5	0 (0)	14 (19.4)	35 (48.6)	22 (30.6)	1 (1.4)
L5/S1	0 (0)	27 (37.5)	23 (31.9)	19 (26.4)	3 (4.2)
Intervertebral disc height, mm					
L1/2	9.7 (1.7)				
L2/3	11.1 (2.0)				
L3/4	11.8 (2.3)				
L4/5	11.4 (2.6)				
L5/S1	10.2 (3.0)				
Chronic low back pain grade, n (%)					
0 – Pain free	14 (19.2)				
1 – Low disability, low intensity	44 (60.3)				
2 – Low disability, high intensity	5 (6.8)				
3 – High disability, moderately limiting	5 (6.8)				
4 – High disability, severely limiting	5 (6.8)				
High intensity pain and or disability, n (%)	15 (20.5)				

Results presented as mean (standard deviation) unless otherwise stated

**Fig. 2:**
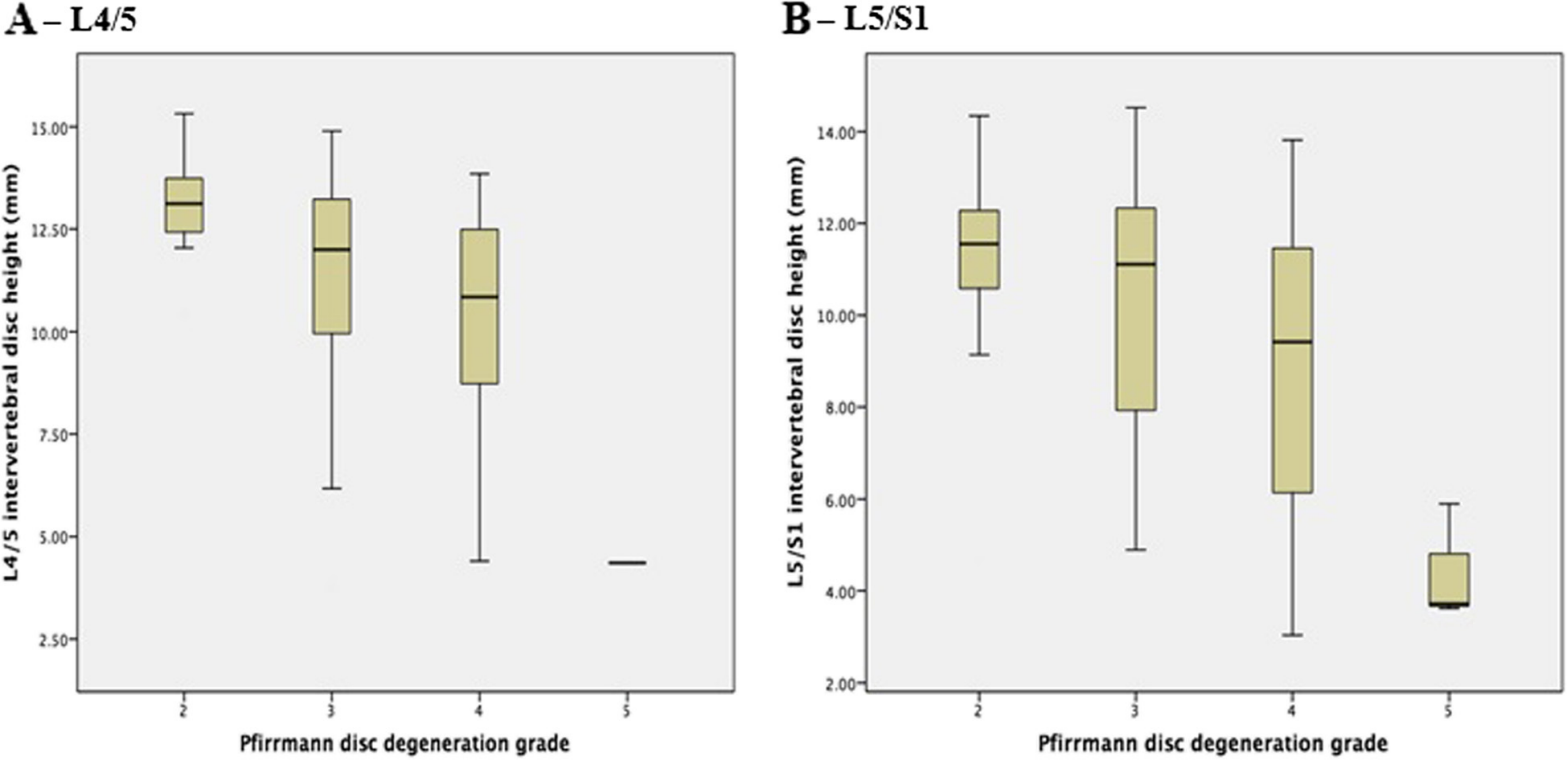
Examples of the relationship between the estimated marginal mean disc height (*Y axis*) and Pfirrmann grade (*X axis*). **a** Vertebrae L4/5. **b** Vertebrae L5/S1

Examples of box plots examining the relationship between the estimated marginal mean for intervertebral disc height (Y axis) and the Pfirrman grade of disc degeneration (X axis) are shown in Fig. 2, while the estimated marginal means of intervertebral disc height according to each grade of disc degeneration at the corresponding lumbosacral level are shown in Table 2. At each level, intervertebral disc narrowing occurred with increasing grades of disc degeneration (all *p* ≤0.003), adjusted for age, gender, BMI and smoking history. To ensure that the significant differences were not driven by grade-5 disc degeneration, which necessitated a collapsed disc space, subgroup analyses were performed with grade-5 disc degeneration excluded. Results remained unchanged (for grades 2–4 disc degeneration, *p* ≤0.03). To further quantify these associations, multiple linear regression analyses were performed (Table 3). For each grade increase in disc degeneration, there was a significant reduction in intervertebral disc height at each lumbosacral level, adjusted for age, gender, BMI and smoking history (all *p* ≤0.009) (L1/2: *β* −0.98 mm (95 % CI −1.65 to −0.31), *p* = 0.005; L2/3: *β* −1.38 mm (95 % CI −2.07 to −0.69), *p* <0.001; L3/4: *β* −1.60 mm (95 % CI −2.37 to −0.83), *p* <0.001; L4/5: *β* −1.36 mm (95 % CI −2.32 to −0.39), *p* = 0.006; L5/S1: *β* −1.21 mm (95 % CI −2.10 to −0.32), *p* = 0.009).

**Table 2:** Estimated marginal means of intervertebral disc height for each grade of disc degeneration

	Intervertebral disc height in mm for each grade of disc degeneration					
	2	3	4	5	*P* ^a^	*P* ^b^
L1/2	10.1 (0.2)	8.9 (0.3)	9.0 (1.0)	3.0 (1.4)	<0.001	0.01
L2/3	12.0 (0.3)	10.5 (0.3)	9.3 (0.6)	-	0.001	0.001
L3/4	13.2 (0.4)	11.4 (0.3)	10.1 (0.6)	5.1 (2.0)	<0.001	0.001
L4/5	13.2 (0.7)	11.5 (0.4)	10.3 (0.5)	4.2 (2.4)	0.003	0.02
L5/S1	11.4 (0.6)	10.5 (0.6)	9.0 (0.6)	4.3 (1.5)	<0.001	0.03

Estimated marginal means (standard error of the mean) adjusted for age, gender, body mass index and smoking history (past/present or never). *P* values for *F* test for pairwise comparison. ^a^Comparisons made for intervertebral disc height for grades 2 to 5. ^b^Comparisons made for intervertebral disc height for grades 2 to 4

**Table 3:** Associations between increasing grade of disc degeneration (grades 2 to 4) and intervertebral disc height in mm at the corresponding level

	Univariate *β* (95 % CI)	*P*	Multivariate^a^ *β* (95 % CI)	*P*
L1/2	−0.98 (−1.61, −0.36)	0.002	−0.98 (−1.65, −0.31)	0.005
L2/3	−1.53 (−2.09, −0.96)	<0.001	−1.38 (−2.07, −0.69)	<0.001
L3/4	−1.61 (−2.26, −0.96)	<0.001	−1.60 (−2.37, −0.83)	<0.001
L4/5	−1.32 (−2.10, −0.54)	0.001	−1.36 (−2.32, −0.39)	0.006
L5/S1	−1.23 (−2.00, −0.46)	0.002	−1.21 (−2.10, −0.32)	0.009

^a^Adjusted for age, gender, body mass index and smoking history (past/present or never)

Post-hoc analyses were performed to compare the disc height for people with and without high pain and/or disability (Table 4). At each individual lumbar spinal level, there was a consistent direction for the intervertebral disc height to be smaller among people with greater pain and or disability, compared to those without. This was statistically significant at the levels of vertebrae L3/4 (10.2 mm vs 12.1 mm, *p* = 0.004) and L4/5 (10.8 mm vs 11.5 mm, *p* = 0.03), as was the cumulative disc height (49.9 mm vs 55.2 mm, *p* = 0.02).

**Table 4:** Average intervertebral disc height in mm (standard error of the mean) according to the presence or absence of high pain and or disability

	No/low pain and disability	High pain and or disability	*P*
	*n* = 57 (79.5 %)	*n* = 15 (20.5 %)	
L1/2	9.9 (0.2)	9.1 (0.4)	0.12
L2/3	11.3 (0.3)	10.3 (0.5)	0.07
L3/4	12.1 (0.3)	10.2 (0.6)	0.004
L4/5	11.5 (0.3)	10.8 (0.7)	0.03
L5/S1	10.4 (0.4)	9.4 (0.8)	0.26
Cumulative L1-S1 disc height	55.2 (1.1)	49.9 (2.1)	0.02

## Discussion

This study has demonstrated a dose–response relationship between the severity of disc degeneration and intervertebral disc height in the lumbosacral spine. In particular, for every grade increase in disc degeneration, there was an associated 0.98 mm to 1.60 mm range of disc narrowing. Although assessment of disc degeneration incorporates a number of qualitative measures, these data substantiate the utility of intervertebral disc height as a quantitative and continuous outcome measure in epidemiological studies.

This study employed the Pfirrmann grading system, a valid and reliable means of assessing intervertebral disc degeneration from MRI, to classify disc degeneration [8, 9]. Descriptors used to assess disc height in the Pfirrmann grading system include normal (grades 1 and 2), normal to slightly decreased (grade 3), normal to moderately decreased (grade 4) and collapsed (grade 5). None of these measures directly quantify disc height, and defining what constitutes normal disc height is ambiguous both within and between individuals. In the current study, we have assessed intervertebral disc height as a continuous measure and demonstrated that for each grade increase in the severity of disc degeneration, there was 0.98 mm to 1.60 mm of disc narrowing at the various levels within the lumbosacral spine, independent of age, gender, BMI and smoking history.

Although the results of this study seem intuitive given that the Pfirrmann grading system incorporates a qualitative assessment of disc height, the only grade to incorporate a marked reduction in disc height is grade 5 (i.e., collapsed disc space). This is reflected by the results of this study, where for instance at the L4/5 level, the estimated marginal mean for intervertebral disc height was 10.3 mm for grade-4 and 4.2 mm for grade-5 disc degeneration. Our results therefore corroborate a discernible difference in disc height between grade-4 and grade-5 disc degeneration. Moreover, at the same level (vertebrae L4/5), the estimated marginal means of intervertebral disc height were 13.2 mm, 11.5 mm and 10.3 mm for grades 2, 3 and 4 disc degeneration, respectively. Such a small magnitude of disc narrowing is unlikely to be qualitatively discernible to assessors for distinguishing an increasing severity of disc degeneration. However we have demonstrated that for each grade increase in disc degeneration (grades 2 to 4) there was significant disc narrowing at each lumbosacral level (all *p* ≤0.03). In a previous study we have demonstrated that reduced intervertebral disc height in the lumbar spine was associated with an increased risk of low back pain [12]. We corroborate this in the current study, showing that intervertebral disc height was smaller among people with greater pain and or disability compared to those without. Similarly, a case–control study of older adults with and without chronic low back pain found that people with more severe degenerative disc disease had a two-fold increased risk of chronic low back pain [2], a finding that has been substantiated elsewhere [3]. Taken together, these data indicate that although both disc degeneration and intervertebral disc height likely measure similar constructs, intervertebral disc height, being a continuous measure, shows concurrent validity in relation to disc degeneration and may provide a more sensitive means for understanding the pathogenesis of lumbosacral disc degeneration.

This study has only examined the Pfirrmann grading to assess disc degeneration. In a systematic review of existing grading systems for lumbar disc degeneration, Pfirrmann’s classification (2001) [8] was endorsed as a valid and reliable method of assessing the intervertebral disc degeneration using MRI [9]. Comparison of these results with other classification systems for assessing disc degeneration would help to substantiate our findings. Moreover, there have been concerns that the Pfirrmann grading system has difficulty discriminating disc pathology in the elderly spine (mean age 73 years; range 67 to 83 years), requiring a modified grading system [13]. Our cohort was younger with a mean age of 48.7 (±8.3) years, therefore averting the need for any modification of the grading system. We have also recruited community-based participants, not selected on the basis of having low back pain. We were concerned that a recruitment strategy necessitating low back pain may have inadvertently led to selection bias towards more severe disc degeneration, thus limiting the generalizability of our findings. Finally, we have noted that grade-5 disc degeneration, which has a hallmark feature of a collapsed disc space, could have been driving the results of this study. However, when we performed subgroup analyses with grade-5 disc degeneration excluded, the results were unchanged. It is also important to acknowledge the different statistical approaches adopted by this study. We employed the *F* test to determine whether the intervertebral disc height for varying grades of disc degeneration was homogenous. This conservative approach cannot, however, discern the direction of these differences. This was quantified in linear regression analyses (Table 3) and demonstrated graphically (Fig. 2). We forced the increment to be the same when moving up one category and treated the score (grades 2, 3 and 4) as a continuous variable.

## Conclusion

This study has demonstrated a dose–response relationship between the severity of disc degeneration and intervertebral disc height in the lumbosacral spine. Although assessment of disc degeneration incorporates a number of qualitative measures, including disc narrowing, these data substantiate the utility of intervertebral disc height as a quantitative and continuous outcome measure in epidemiological studies.

## Abbreviations

BMI: body mass index
CV: coefficient of variation
ICC: intra-class correlation coefficient
MRI: magnetic resonance imaging
